# Anti-Inflammatory Potential of Fucoidan for Atherosclerosis: In Silico and In Vitro Studies in THP-1 Cells

**DOI:** 10.3390/molecules27103197

**Published:** 2022-05-17

**Authors:** Etimad Huwait, Dalal A. Al-Saedi, Zeenat Mirza

**Affiliations:** 1 Department of Biochemistry, Faculty of Sciences, King Abdulaziz University, Jeddah 21589, Saudi Arabia; dalal.alsaadi@hotmail.com; 2 Cell Culture Lab, Experimental Biochemistry Unit, King Fahd Medical Research Centre, King Abdulaziz University, Jeddah 21589, Saudi Arabia; 3 King Fahd Medical Research Center, King Abdulaziz University, Jeddah 21589, Saudi Arabia; 4 Department of Medical Laboratory Sciences, Faculty of Applied Medical Sciences, King Abdulaziz University, Jeddah 21589, Saudi Arabia

**Keywords:** fucoidan, alginate, L-selectin, E-selectin, MCP-1, ICAM-1, molecular docking, THP-1 macrophage, monocyte migration

## Abstract

Several diseases, including atherosclerosis, are characterized by inflammation, which is initiated by leukocyte migration to the inflamed lesion. Hence, genes implicated in the early stages of inflammation are potential therapeutic targets to effectively reduce atherogenesis. Algal-derived polysaccharides are one of the most promising sources for pharmaceutical application, although their mechanism of action is still poorly understood. The present study uses a computational method to anticipate the effect of fucoidan and alginate on interactions with adhesion molecules and chemokine, followed by an assessment of the cytotoxicity of the best-predicted bioactive compound for human monocytic THP-1 macrophages by lactate dehydrogenase and crystal violet assay. Moreover, an in vitro pharmacodynamics evaluation was performed. Molecular docking results indicate that fucoidan has a greater affinity for L-and E-selectin, monocyte chemoattractant protein 1 (MCP-1), and intercellular adhesion molecule-1 (ICAM-1) as compared to alginate. Interestingly, there was no fucoidan cytotoxicity on THP-1 macrophages, even at 200 µg/mL for 24 h. The strong interaction between fucoidan and L-selectin in silico explained its ability to inhibit the THP-1 monocytes migration in vitro. MCP-1 and ICAM-1 expression levels in THP-1 macrophages treated with 50 µg/mL fucoidan for 24 h, followed by induction by IFN-γ, were shown to be significantly suppressed as eight- and four-fold changes, respectively, relative to cells treated only with IFN-γ. These results indicate that the electrostatic interaction of fucoidan improves its binding affinity to inflammatory markers in silico and reduces their expression in THP-1 cells in vitro, thus making fucoidan a good candidate to prevent inflammation.

## 1. Introduction

Inflammation is the prime cause of cardiovascular diseases (CVDs), one of the most frequent reasons of death worldwide [1]. Atherosclerosis is one of the most frequent CVDs; it is an arterial hardening and subsequent narrowing caused by lipid deposition and gradual plaque buildup within an artery wall. This complex disease is initiated by inflammation and often leads to a stroke or heart attack [2]. The pathogenesis of atherosclerosis starts with the interaction between leukocytes and endothelium, followed by immune cell migration to the inflammatory lesion in a multi-step process called extravasation monocyte, involving adhesion and signaling molecules such as selectins and chemokines. This is characterized by tethering, the rolling of monocytes on vascular surfaces of endothelium, tight adhesion, and migration during inflammation [3].

Selectins (L- and E-selectin) are transmembrane receptors, which are expressed on leukocytes and activated endothelial cells, respectively. Their function is based on the extracellular lectin domain’s calcium-dependent interaction with Lewisx sialyl (sLex) tetrasaccharide expressed on the glycoprotein [4,5], which mediates the initial stage of cell adhesion on the endothelial cell surface. Endothelial activation in response to proinflammatory cytokines secretes chemokines such as monocyte chemoattractant protein 1 (MCP-1 aka C-C motif chemokine 2, CLL2), which activates the C-C chemokine receptor type 2 (CCR2) on monocyte, followed by stimulating integrin β2 to a high-affinity state, enabling it to bind to intercellular adhesion molecule-1 (ICAM-1), which is overexpressed due to endothelial dysfunction [3,6]. The mechanism that hinders monocyte migration through blocking inflammatory biomarkers is critical to the early halting of inflammation.

Glycosaminoglycans (GAGs) are negatively charged linear polysaccharide chains that are covalently bound to proteins, for example, dermatan sulfate, heparan sulfate, and chondroitin sulfate, which mediate significant physiological functions, for instance, inflammation, through signaling and recognition [7,8,9,10]. Marine natural products are a promising therapeutic source of bioactive compounds. The polysaccharides derived from macroalgae have gained worldwide attention due to their myriad of structural, physicochemical, and biological activities. Fucoidan is a sulfated polysaccharide, which is mostly built from sulfated L-fucose molecules and other monomeric sugars, such as glucose, galactose, mannose, and uronic acid. Alginates are natural linear copolymers of α-L-guluronic acid and β-D-mannuronic acid, which widely exist in brown seaweeds [11,12]. The structure–activity relationship of fucoidan affects diverse biological activities; indicating promising pharmacological potential [13,14,15,16], although the molecular mechanism is still unknown. A recent study indicates that fucoidan extracts from different algal species, including *Fucus vesiculosus*, reduce the inflammatory cytokine levels in lipopolysaccharide-stimulated peripheral blood mononuclear cells and leukemia monocytic cell line (THP-1) in a dose-dependent fashion [17]. Moreover, fucoidan from *Fucus vesiculosus* inhibits lung cancer cell migration and invasion via phosphatidylinositol-3-kinase (PI3K)/Akt and the mammalian target of rapamycin (mTOR) signaling [18].

The computational prediction of the interaction between bioactive compounds and therapeutic target proteins rationally guides experimental methods and significantly reduces the cost of drug development [19]. Therefore, we predict and compare the ability of sulfated (fucoidan) and non-sulfated (alginate) polysaccharides to computationally interact with several targets that are implicated in inflammation, particularly endothelial dysfunction and monocyte migration, including L-selectin, E-selectin, MCP-1, and ICAM-1, by using the most frequently occurring monomer in polysaccharides. We also explored the pharmacodynamics of fucoidan on the above-mentioned inflammatory markers in THP-1 cells.

## 2. Results

**Figure 1:**
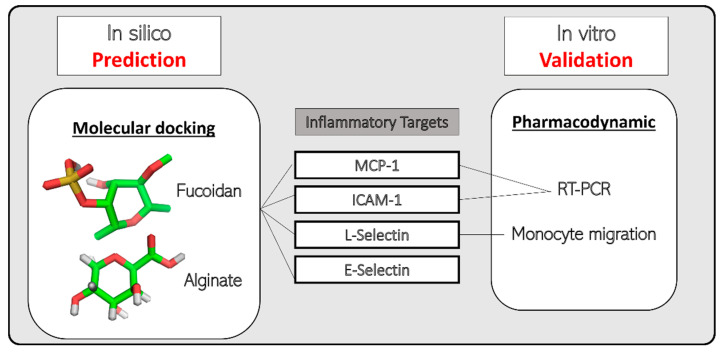
Study approach for prediction and validation of marine bioactive compounds (fucoidan and alginate) with inflammatory protein targets.

This study aimed to understand the interaction between fucoidan and alginate with the potential inflammatory biomarkers (L-selectin, E-selectin, MCP-1, and ICAM-1, respectively) that are vital in monocyte migration. Firstly, the PPIs were predicted, followed by docking, to illustrate protein–ligand interactions. Finally, the best prediction was validated using experimental methods. Figure 1 summarizes our study approach.

### 2.1. Protein–Protein Interaction

**Figure 2:**
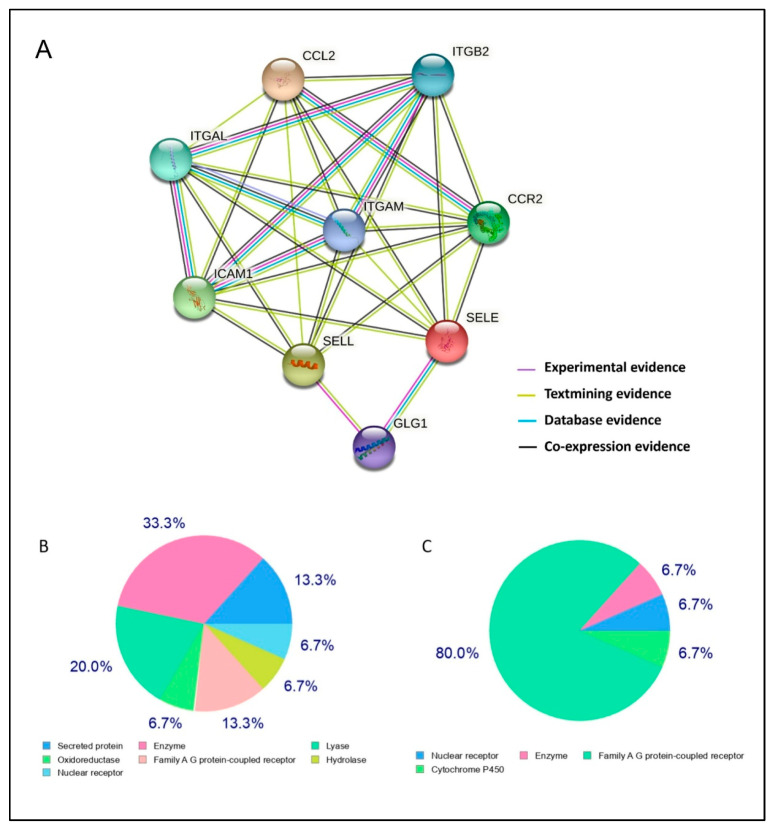
Prediction of biomolecular interactions. (**A**): STRING protein–protein interaction (PPI). Nodes in the network represent proteins, and different types of interaction evidence are indicated by interconnecting colored lines (co-occurrence: blue; purple; experimental: purple; text-mining: yellow; database: light blue; co-expression: black). (**B**,**C**): Swiss Target Prediction of the top 15 target categories for marine bioactive compounds: fucoidan and alginate, respectively.

A summarized network of the predicted associations for inflammatory proteins is illustrated in Figure 2. Interestingly, the ICAM1 protein could be linked to seven predicted functional partners, namely, L-selectin (SELL), E-selectin (SELE), C-C chemokine receptor type 2 (CCR2), C-C motif chemokine 2 (CCL2), integrin subunit β2 (ITGB2), integrin α-M (ITGAM), and integrin αl (ITGAL). As such, all proteins were directly related to SELL, except for SELE, which was indirectly linked through GLG1. Integrin α-M acts as the network hub, while the evidence of co-expression showed the most associations between the above-mentioned protein entities.

### 2.2. Chemoinformatic Analysis

**Figure 3:**
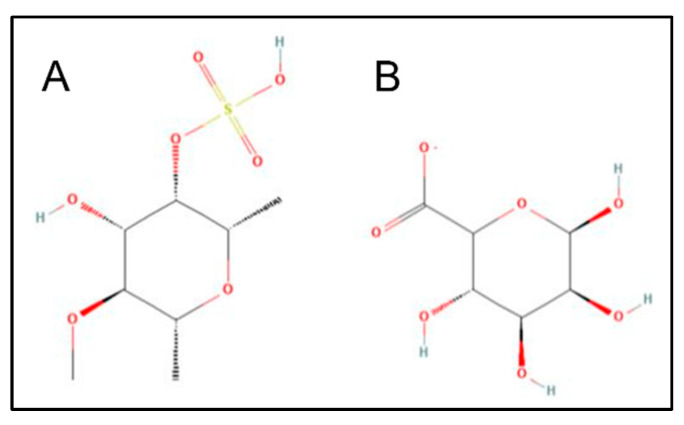
2D chemical structure of fucoidan (**A**) and alginate (**B**).

The ligand-based target prediction of classified targets fucoidan and alginate molecules by SwissTargetPrediction tool (Figure 2B,C) shows seven and four classes of human proteins, respectively. Alginate most likely interacts with G-protein coupled receptors (GPCRs) as (80%), whereas fucoidan can trigger GPCRs and secreted proteins at a similar rate of 13.13%. Physicochemical properties have great significance from the perspective of the medicinal chemistry of the drug development process. The SwissADME analysis for fucoidan and alginate monomer illustrates that both are hydrophilic (MLog *p* = −1.49 and −2.89, respectively). They have same number of hydrogen bond acceptors and a different number of hydrogen donors and rotatable bonds (Table 1). The 2D chemical structure of both the dietary ligands is shown in Figure 3.

### 2.3. Molecular Docking and Potential Binding Site Prediction

**Figure 4:**
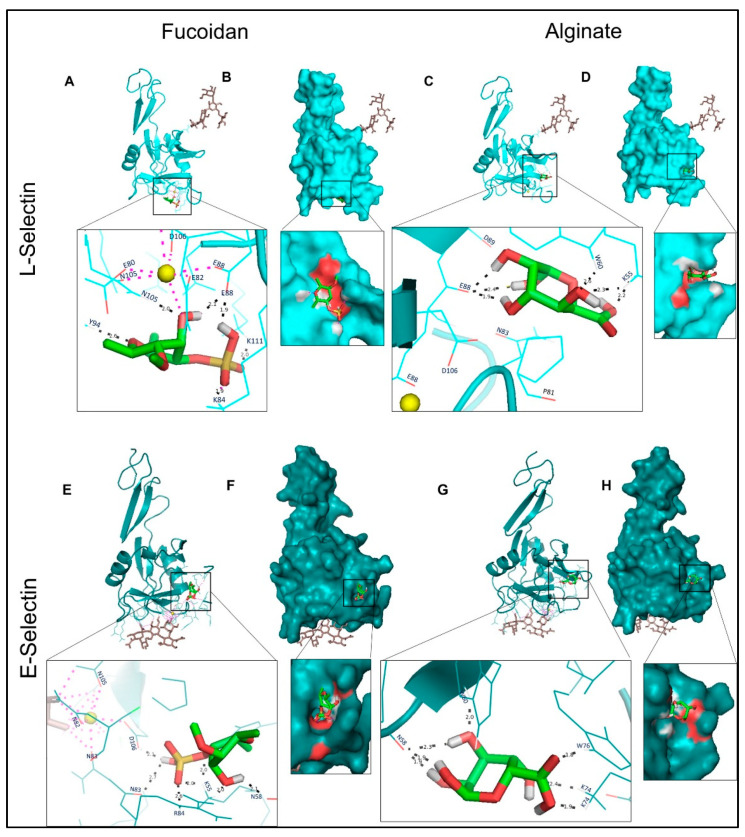
Molecular docking of L-and E-selectins with fucoidan and alginate. (**A**,**C**,**E**,**G**): 3D structures of proteins bound with N-linked glycan moieties (brown) and zoomed ligand-binding pocket. Black and purple dotted lines, respectively, describe the H-bonds and Ca^2+^ (yellow ball) coordination bonds. (**B**,**D**,**F**,**H**): molecular surface representation, and the red patches on the surface represent electrostatics of the binding cavity.

Molecular docking prediction was carried out to estimate binding affinity of fucoidan and alginate with target proteins, considering root mean square deviation <2Å. The lower binding energy corresponds to the higher affinity of the protein–ligand complex. As compared to alginate, fucoidan shows the higher binding affinity and lowest inhibition constant to the four target inflammatory proteins, as summarized in Table 2. The estimated free energy for the binding of L-selectin to fucoidan was −5.82 kcal/mol via the hydrogen bonds formed with Lys48 (1.9Å), Asn105 (2.0Å), Lys111 (2.0Å), and two bonds with Glu88 (1.9 and 2.0Å) in coordination with Ca^2+^ in the putative binding site (Figure 4A). In contrast, alginate binds via two hydrogen bonds with Glu88 (2.4 and 1.9Å) residues of L-selectin (Figure 4C) in a different position compared to fucoidan, without coordination with Ca^2+^, resulting in a lower binding energy (−4.3 kcal/mol), and exhibit lesser electrostatic interactions, as shown in Figure 4B,D by the red region of the molecular surface. Based on energy and binding affinity values, the interaction of fucoidan with E-selectin is superior to that of alginate. The sulfate groups in fucoidan bind with the Asn83 (2.71Å) and Asp106 (2.06Å) residues at the carbohydrate recognition site in coordination with Ca^2+^ (Figure 4E). Stronger hydrogen bonds are formed by alginate without the assistance of Ca^2+^ (Figure 4G).

**Figure 5:**
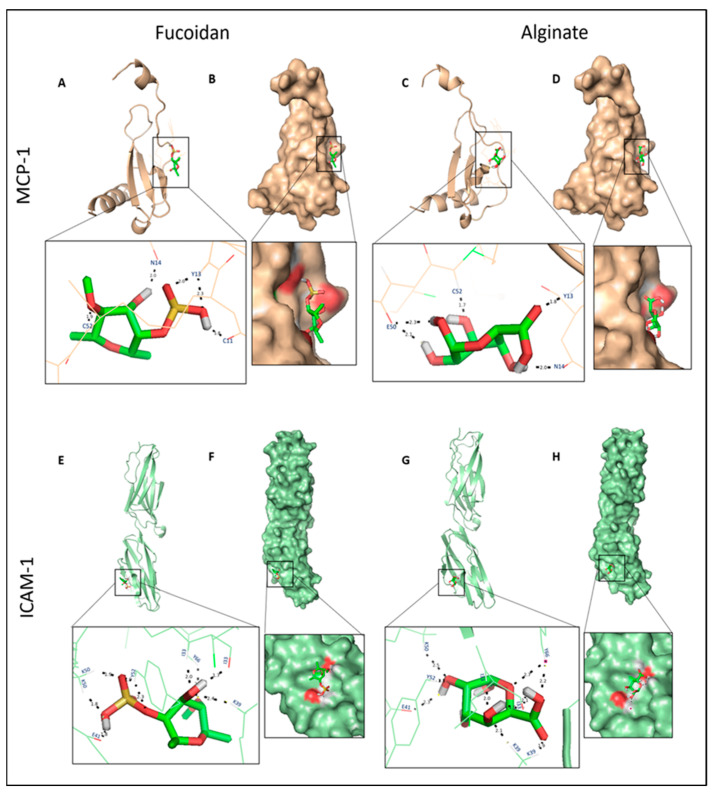
Molecular docking of MCP-1 and ICAM-1 to fucoidan and alginate. (**A**,**C**,**E**,**G**): 3D structures of proteins bound and ligand-binding pocket. Hydrogen bonds is represented by black dotted lines. (**B**,**D**,**F**,**H**): molecular surface representation, and the red patches on the surface represent electrostatic binding pocket.

During molecular visualization, it was observed that fucoidan docked to MCP-1 within the N-loop (Tyr13 and Asn14) and β3-strand (Cys11 and Cys52) with an estimated free binding energy of −5.67 kcal/mol. Alginate binds with similar residues of MCP-1, except that the interactions between Cys 11 and Tyr13 are not seen; instead H-bonds with Glu50 are noticed, which decreases the binding energy to −3.84 kcal/mol (Figure 5A–D). ICAM-1 non-covalently interacts with fucoidan and alginate through similar residues (Lys50, Lys39, Tyr66, Tyr52, Glu41, and Leu33) but short-distance hydrogen bonds in the binding site. Fucoidan’s binding interaction energy was observed to be −5.66 kcal/mol (Figure 5E–H). Based on in silico data, fucoidan was chosen to understand the nature of interactions with selected inflammatory markers (L-selectin, MCP-1, and ICAM-1) in further in vitro studies.

### 2.4. Effect of Fucoidan on Viability and Proliferation of THP-1 Macrophages

**Figure 6:**
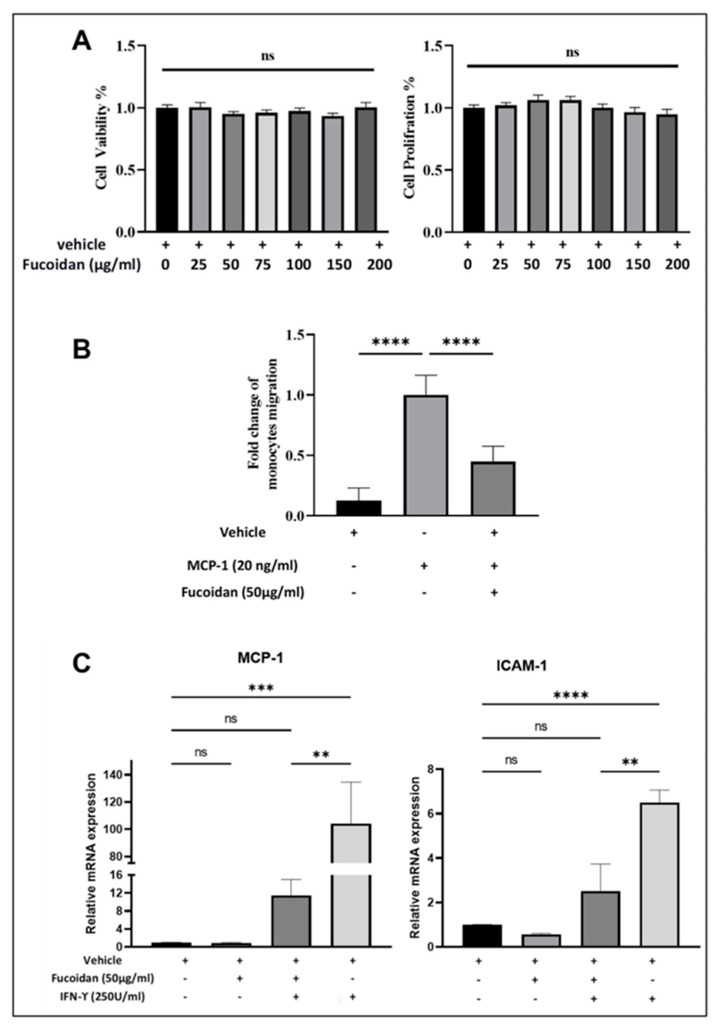
Biological activity of fucoidan on THP-1 cells. (**A**): Percentages of cell viability and proliferation on THP-1 macrophages subjected to various concentrations of fucoidan exposed for 24 h. (**B**): THP-1 monocyte migration assessed using a transwell chamber after 3 h stimulation with or without MCP-1 in the presence or absence of vehicle or fucoidan (50 µg/mL). (**C**): mRNA expression level of MCP-1 and ICAM-1 evaluated in THP-1 macrophages post treatment with fucoidan (50 µg/mL), vehicle, or alone for 24 h. Then cells were induced with or without IFN-γ for 3 h. Data were presented as mean ±SEM of triplicate three/two independent experiments (*n* = 9 for (**A**,**B**) and *n* = 6 for (**C**) and the *p*-values were non-significant (ns), ** *p* < 0.001, *** *p* < 0.0005 and **** *p* < 0.0001.

To evaluate the effects of fucoidan on cell viability, an LDH assay was carried out, and the results were validated by assessing cell proliferation with crystal violet. Figure 6A demonstrates that fucoidan does not pose significant cytotoxicity to THP-1 macrophages when treated with increased doses compared to the vehicle. A total of 50 µg/mL fucoidan was chosen for further experiments in accordance with the published literature [20,21,22].

### 2.5. Fucoidan Inhibits Monocytes Migration to MCP-1

As shown in Figure 6B, the migration of THP-1 monocytes significantly increased in the presence of MCP-1 alone compared to vehicle, while the percentage migration of cells treated with fucoidan was significantly attenuated, by 50%, in response to chemokine MCP-1.

### 2.6. Fucoidan Modulates the Expression of Inflammatory Markers

MCP-1 and ICAM-1 are critical inflammatory genes for endothelium dysfunction. The transcriptomics of these genes in THP-1 macrophages, and their post-treatment with fucoidan and IFN-γ, is illustrated in Figure 6C. MCP-1 transcription was dramatically decreased by eight-fold in cells treated with IFN-γ in the presence of fucoidan compared to cells treated with IFN-γ alone. Interestingly, the effects of vehicle and fucoidan alone on the expression of both genes are not significantly different. Regarding the expression levels of ICAM-1 in THP-1 macrophages, fucoidan can attenuate the IFN-γ induced ICAM-1 expression in THP-1-derived macrophages by four-fold.

## 3. Discussion

Preventing leukocytes recruitment to inflammation sites can address the early stage of atherosclerosis, which is predominantly mediated by L-selectin [23,24]. L-selectin has a high affinity for binding sulfated carbohydrate moieties on *p*-selectin glycoprotein ligand-1 (PSGL-1), a glycoprotein located on leukocytes and endothelial cells that naturally binds to the selectin family. Upon the activation of endothelium, transcription-regulated E-selectin mediates the adhesion of neutrophils via PSGL-1 [25,26,27] or Golgi apparatus protein 1 (GLG1) within hours [28]. Endothelial activation triggers a chronic inflammatory response that involves the release of MCP-1, which subsequently binds and activates CCR2, the GPCRs embedded in the leukocytic cell membranes [29]. The signal transduction of the chemokine receptors initiates signaling to activate integrins, which are transmembrane heterodimeric proteins comprised of α and β subunits and responsible for firm adhesion to the extracellular matrix (ECM) and regulating the ‘inside-out’ cellular signaling. High-affinity integrins enable the tight adhesion of ICAM-1 to the transmigration of leukocytes through vascular endothelium [3,6]. Blocking these inflammatory biomarkers is crucial to stop or reduce atherosclerosis.

Polysaccharides are natural macromolecular polymers that can be found in a variety of dietary sources and have attracted a great deal of attention due to their important bioactivities [8]. The negative sulfate charges are known to play a role in the electrostatic interactions between GAGs and signaling proteins [10]. Fucoidan is a class of sulfated, fucose-rich polysaccharides present in diverse species of brown seaweed. Its unique features make it a promising candidate for nutraceuticals and pharmaceuticals for disease prevention [16,17,21]. Owing to the variety of chain structures, sulfation degrees, and positions, the structure–activity relationship between fucoidan and its mechanism of action is challenging to understand [9,17,21,22]. Therefore, we investigated the pharmacodynamics of fucoidan derived from *Fucus vesiculosus* as having anti-inflammatory potential for atherosclerosis on THP-1 cells.

Our results indicate that fucoidan has no significant cytotoxic effects on THP-1 macrophages, even at 200 µg/mL, which is consistent with several studies that examine cytotoxicity for 72 h [20,21,22]. Furthermore, molecular docking shows that, when fucoidan occupies the binding site in inflammatory proteins, it prevents the interaction between these proteins and other downstream regulatory partners and perturbs signaling. For instance, we found that it inhibits L-selectin, which is responsible for the adhesion of leukocytes, and suppresses MCP-1 and ICAM-1.

Hydrophilic drugs are desired for oral administration due to their bioavailability and easy formulation [30]. Lipinski’s rule helps to estimate a compound’s drug-likeness and includes molecular weight < 500 Da; LogP < 5; hydrogen-bond donors < 5 and hydrogen-bond acceptors < 10 [19]. The physiochemical features of both the ligands comply with these features. Moreover, the sulfated hydroxyl group imposes steric effect changes and electrostatic repulsion, causing flexion and extension of the polysaccharide chain and increased hydrophilicity, leading to improved affinity with proteins, and thereby altering biological activities [13,31]. Another indicator of a compound’s flexibility is presence of rotatable bonds [32]. Our computational predictions refer to fucoidan’s higher affinity for target proteins compared to alginate due to the flexibility that results from three rotatable bonds. Notably, the number of hydrogen bonds predicted via the Swiss tool was in accordance with the molecular docking of inflammatory proteins with selected ligands.

The electrostatic interactions of fucoidan most probably play a role in aiding the sulfate group’s binding to Lys84 on L-selectin, similar to negatively charged Tyr51 of PSGL-1, which has 6-sulfo-sLex binding to L-selectin Lys85 [33]. The native binding site of selectins with Ca^2+^ in the lectin domain has identical residues, namely, Glu80, Glu88, Asn82, Asn105, and Asp106. This binding is explained by two conformations: it is extended with Asn83 coordinating Ca^2+^ and Glu88 away or bent with Glu88 coordinating Ca^2+^ and Asn83 away. This leads to a structural change that affects the re-orientation of the lectin and EGF-like domains, thereby stabilizing the high-affinity ligand-bonded state, which is vital to enduring the shearing force in the bloodstream and makes rolling less stable [34]. As shown in Figure 3A, fucoidan binds with Glu88, in coordination with Ca^2+^ molecule. It is worth mentioning that a comparative anti-inflammatory and anti-adhesive study investigated the origin and composition of fucoidans from diverse algal species, indicating that specific structural motifs of the fucoidans might mimic SLeX, resulting in suppressed L-selectin [35]. Our in silico results display that fucoidan binds strongly to L-selectin active sites. This supports the experimental findings of an inhibitory effect on the migration of THP-1 monocytes and suggests that fucoidan could be an antagonist for L-selectin, as previously mentioned [36]. A recent report also indicates that targeting L-selectin holds promise to control inflammation [37].

Residues in the alternative inflammatory target MCP-1’s N-loop and B3 domain are necessary for binding interactions, while residues in the N-terminal area are important for receptor activation, according to structural–functional studies of chemokines [38,39]. To better understand the contribution of selective binding and activation by chemokine proteins to the chemokine receptor CCR2, Huma et al. assessed the binding of chemokine structure regions to CCR2 and observed that the N-terminal of chemokine is a major determinant of affinity and efficacy [29]. They postulated that chemokines attach to the receptor N-terminus via their N-loop and β3 residues (site1), and then the chemokine N-terminus (site2) activates the receptor by binding to its transmembrane helices, producing conformational changes and cellular signaling. Both bioactive compounds in this study bind between N-loop and β3 regions and could compete for CCR2 and obstruct binding. The results of other comparative study indicate that the hydroxyl groups of three types of flavanols (kaempferol, quercetin, and myricetin, respectively) bind with MCP-1 (−5.10, −5.28, and −6.39 kcal/mol, respectively) via common residues Cys11, Cys52, Asn14, Tyr13, and Lys16, which overlapped with that of the receptor-GAG-binding surface, hence indicating that chemokine-mediated leukocyte trafficking is likely reduced [40]. Although fucoidan and alginate both bind MCP-1 with same residues, alginate binds with a lower binding energy of −3.84 kcal/mol, while the sulfate group in fucoidan enhances this binding energy to −5.67 kcal/mol. The treatment of THP-1 macrophages with fucoidan for 24 h stimulates them to create inflammatory cytokines induced by IFN-γ, a macrophage-activating factor, as previously reported [41]. Fucoidan, hence, offers protective effect by drastically reducing MCP-1 expression.

The integrin’s I domain-binding surface of ICAM-1 is relatively shallow, and Glu34 is present in the middle of the ICAM-1 coordination bond, with an Mg^2+^ ion in the I domain [42]. Furthermore, aromatic and hydrophobic residues on the ICAM-1 surround Glu34, Pro36, Tyr66, Met64, and the aliphatic portions of Gln62 and Gln73 contact the similar ring of hydrophobic residues on the I domain [43]. As a result, the electrostatic surface’s contact regions have good charge complementarity. For ligand binding, a salt bridge between the I domain Glu241 and ICAM-1 Lys39 is required, allowing for ICAM-1 and the I domain to optimally interact [44,45]. Similar residues, including Lys39, were found in our study, participating in interactions with both ligands and ICAM-1. Polar interactions involving hydrogen bonds sustain this interaction, which is shorter in fucoidan, possibly due to its greater negative charges. Although MCP-1 and ICAM-1 have a similar binding affinity to fucoidan in terms of docking results, fucoidan suppresses ICAM-1 expression in THP-1 macrophages that undergo IFN-γ induction, with a lower fold change than MCP-1, which means fucoidan interacts with the non-specific protein [46]. Moreover, anionic polysaccharide can bind to distinct proteins with several levels of specificity to endothelial cells [47].

Even though this study lacks protein expression evaluation, an understanding of docking interactions with fucoidan and validated with gene expression experiments helps us gain knowledge of the effect at the protein level. It is worth mentioning that fucoidan can inhibit these proteins at 55–70 µM, according to the predicted inhibition values that are constant in molecular docking. That implies only a small amount is required to inhibit the protein’s activity.

## 4. Materials and Methods

### 4.1. Protein–Protein Interaction Study

The significant protein–protein interactions (PPIs) existing between L-selectin, E-selectin, MCP-1, and ICAM-1 were explored using STRING protein database version 11.5. Network edges (evidence), and active interaction sources (text mining, databases, experiments, neighborhood, co-expression) were then employed as the primary settings, and limited to homo sapiens. Minimal required interaction score of >0.4 was applied to construct the PPIs networks [48].

### 4.2. Chemoinformatic Prediction

Chemoinformatic tools were employed to predict suitability of bioactive molecules (fucoidan and alginate) as a drug. SwissTargetPrediction predicts the most probable protein targets of biomolecules based on a blend of 2D and 3D structural and electrochemical complementarity [49]. SwissADME online tool evaluates physicochemical descriptors by computing the ADME features and drug likeliness of small molecules for consideration as an oral drug candidate [50].

### 4.3. Molecular Docking

Three-dimensional X-ray structures of inflammatory proteins, namely, L-selectin, E-selectin, MCP-1, and ICAM-1, were retrieved from RCSB’s Protein Data Bank (PDB) (ID: 5VC1, 1G1T, 1DOK, and 1IAM, respectively) with a resolution of 1.85Å, 2.1Å, 1.94Å, and 1.5Å, respectively) [5,26,51,52]. The 3D structures of fucoidan and alginate (CID: 129532628 and 91666324, respectively) were downloaded from NCBI’s PubChem database, and protein and ligand structures were prepared, followed by molecular docking to compute the binding energy in kcal/mol resulting from the interaction of fucoidan and alginate with proteins using Auto Dock 4.2.6 [53]. Docking was performed with monomeric unit of polysaccharides. Each protein structure was processed by selecting one chain and removing the water molecules and the existing co-crystallized ligand. The grid dimensions were generated according to the known binding sites of each protein. Docking was protein-rigid and ligand-flexible. Binding free energy of ligand-protein interaction was used to score various configurations. The best pose was chosen based on the lowest docking energy (kcal/mol) and lower RMSD [54]. Complex structures were visualized by PyMol 1. Level (DeLano Scientific LLC., Palo Alto, CA, USA).

### 4.4. Cell Culture

THP-1, a human monocytic leukemia cell line, was provided by Molecular Biomedicine Unit, King Faisal Specialist Hospital and Research Centre, Riyadh, KSA. THP-1 cells were maintained as an undifferentiated monocyte grown in suspension in RPMI medium 1640 (1×) supplemented with fetal bovine serum (FBS, 10% *v/v*), L-glutamine (200 mM, 1% *v/v*) and penicillin-streptomycin (100 U/mL) (Gibco^TM^, ThermoFisherScientific, Waltham, MA, USA). Cell incubation was carried out in an atmosphere with 5% CO_2_, 95% humidity and a 37 °C temperature.

#### 4.4.1. Cell Viability and Proliferation Assays

A lactate dehydrogenase (LDH) cytotoxicity assay was carried out for cell viability measurement following the manufacturer’s instructions (88953; ThermoFisherScientific, Waltham, MA, USA). Seeding of THP-1 monocytes was carried out with a density of 1 × 10^5^ cells/cm^2^ in 96-well plates and differentiation into macrophages was performed with 0.16 µL of phorbol myristate acetate (PMA, 1 mg/mL, ThermoFisher (Kandel) GmbH, Germany) overnight at 37 °C and 5% (*v/v*) CO_2_. Fucoidan (≥95% HPLC, F8190; Sigma-Aldrich, St. Louis, MO, USA) was dissolved in pure distilled water (vehicle) at 10 mg/mL and then diluted in culture media at different concentrations to treat the macrophages for a further 24 h. Subsequently, 50 μL supernatants of treated THP-1 macrophages were transferred into new 96-well plates, along with a 50 μL assay buffer. Following incubation for 30 min at 25 °C, 50 μL of stopping solution was mixed. Absorbance was noted at 490 nm using a microplate reader (BioTek Synergy HT, Agilent Technologies, Santa Clara, CA, USA). Crystal violet dye was used to evaluate the proliferation of cells via binding to the DNA of viable cells [55]. Adherent macrophages remaining after the LDH test were employed for the cell proliferation assay. Cells were stained with 50 µL of 0.2% (*w/v*) crystal violet solution (dissolved in 10% ethanol) for 5 min at room temperature. THP-1 macrophages were washed 3–4 times with PBS prior to the addition of 50 µL of solubilization buffer (0.1 M NaH_2_PO_4_ ethanol solution). Treated plate was shaken for 5 min before measuring absorbance with a microplate reader at 570 nm. Results were tabulated as the percentage of viability related to control.

#### 4.4.2. Migration Assay

Migration assay was used to estimate fucoidan’s ability to inhibit monocyte migration in response to chemoattraction. A 1 mL culture media containing 20 ng/mL of monocyte chemoattractant protein (MCP-1/MCAF, Sigma-Aldrich, St. Louis, MO, USA, SRP3109) was added to the bottom of companion plates of the SPL Insert hanging (35224; SPL Life Sciences, Gyeonggi-do, Korea) in all wells except the control well. Undifferentiated THP-1 monocyte cells (5 × 105 cells/mL) were added to inserts with a 0.8 µm pore size. Then, immediately after being treated with either a control (vehicle) or 50 µg/mL of fucoidan, cells alone were used as a positive control for MCP-1. Plate chambers were incubated with 5% (*v/v*) CO_2_ at 37 °C for 3 h. Cells that had migrated into the lower chambers were collected, and centrifuged at 250× *g* for 5 min. Cell pellets were resuspended in 1 mL of fresh media and cells were counted using a hemocytometer [56]. Monocyte migration was expressed as a fold-change relative to the fraction of cells that moved through the insert into the bottom wells in response to chemokine alone.

#### 4.4.3. Quantitative Reverse Transcription-PCR

Two groups of THP-1 macrophages were taken (untreated and treated with 50 µg/mL fucoidan for 24 h). Inflammation was induced in both the groups with 0.13 µL of interferon-γ human (INF-γ, 13265; 1 mg/mL, Sigma-Aldrich) treated for 3 hrs. Total mRNA extraction was carried out for all (vehicle, fucoidan alone, fucoidan with IFN-γ and IFN-γ) using the RNeasy™ mini kit (74104; Qiagen, Germany) and transcribed into cDNA as per the instructions using the ImProm-II Reverse Transcription kit (A3800; Promega, Madison, WI, USA). A quantitative polymerase chain reaction (qPCR) was performed using the BioFACT^TM^ 2X Real-Time PCR Master Mix (For SYBR^®^ Green I) kit (DQ383–40h; Daejeon, Korea). Target genes (MCP-1 and ICAM-1) expression was analyzed by a StepOnePlusTM Real-time PCR system (Applied Biosystems, Waltham, MA, USA). Relative quantification of their expression with fold change and *p*-value was calculated using the comparative threshold method (Ct, 2–ΔΔCT) after normalization with glyceraldehyde-3-phosphate dehydrogenase (GAPDH) housekeeping gene. Table 3 enlists the primers that were used [56].

#### 4.4.4. Statistical Analysis

Statistical analysis was performed using one-way ANOVA to detect any statistically significant differences between the means of two or more independent groups, followed by a Sidak multiple comparison test. Excel Microsoft 365 and GraphPad Prism version 8 softwares were used for statistical analysis. The significance is represented using *p*-values as ns (non-significant), ** *p* < 0.001, *** *p* < 0.0005 and **** *p* < 0.0001.

## 5. Conclusions

Natural compounds can potentially alter or regulate cellular gene expression, aiding in the treatment and prevention of any diseases hallmarked by inflammation. The pharmacodynamically relevant ability of fucoidan to modulate key biomarker genes in the early stages of atherosclerosis was demonstrated. Fucoidan potentially blocks L-selectin and prevents monocyte migration, thereby modulating the expression level of MCP-1 and ICAM-1 in THP-1 macrophages. Our results support in silico molecular docking results, wherein fucoidan occupies the binding sites of inflammatory proteins. Future in vivo investigations will help us to better comprehend the underlying mechanisms at the molecular level, as well as the anti-inflammatory effects of natural substances and their use as dietary supplements. This emphasizes the benefits of a nutritionally orientated approach to prevent initial disease development. Pre-clinical trials are further needed to determine the efficacy of fucoidan and establish its role in the prevention and treatment of inflammatory disorders, including atherosclerosis.

## Figures and Tables

**Table 1:** Analysis of swissADME for marine bioactive compounds (fucoidan and alginate).

Physicochemical Properties	Fucoidan	Alginate
MLogP	−1.49	−2.89
Molecular weight	256.27	193.13
Number of H-bond acceptors	7	7
Number of H-bond donors	2	4
Number Rotatable bonds	3	1

Note: LogP refers to the octanol–water partition coefficient.

**Table 2:** AutoDock docking results of marine bioactive compounds (fucoidan and alginate) with inflammatory proteins.

Protein	PDB ID	Ligand	Binding Energy (Kcal/mol)	Inhibition Constant (Ki)	Interacting Residues
**L-selectin**	5VC1	Fucoidan	−5.82	54.41 µM	Lys84(1.9Å), Glu88(1.9Å), Tyr94(2.6Å), Asn105 (2.0Å), Lys111(2.0Å),
		Alginate	−4.3	704.72 µM	Lys55(2.2 Å), Trp60(2.6Å), Glu88(1.9 Å),
**E-selectin**	1G1T	Fucoidan	−5.69	67.62 µM	Lys55(2.0Å), Asn58(2.1Å), Asn83(2.7Å), Arg84(2.6Å), Asp106(2.1Å)
		Alginate	−4.09	997.16 µM	Asn58(1.9 Å), Trp60(2.0 Å), Lys74(1.9Å), Trp76(1.8 Å)
**MCP-1**	1DOK	Fucoidan	−5.67	69.96 µM	Cys11(1.8 Å), Tyr13(2.1 Å), Asn14(2.0Å), Cys52(1.9 Å)
		Alginate	−3.84	1.52 mM	Asn14(2.0 Å), Glu50(2.1Å), Cys52(1.7Å)
**ICAM-1**	1IAM	Fucoidan	−5.66	70.39 µM	Leu33(1.7Å), Lys39(2.4Å), Glu41(1.8Å), Lys50(1.8Å), Tyr52(1.9Å), Tyr66(2.1Å)
		Alginate	−4.98	224.33 µM	Leu33(1.9Å), Lys39(1.9Å), Glu41(2.3 Å), Lys50(2.5Å), Tyr52(2.1Å), Tyr66(2.2Å)

**Table 3:** Primer Sequences used for human MCP-1, ICAM-1 and GAPDH genes.

Gene	Primer Sequence
*MCP-1*	Forward: CGCTCAGCCAGATGCAATCAATG Reverse: CGCTCAGCCAGATGCAATCAATG
*ICAM-1*	Forward: GACCAGAGGTTGAACCCCAC Reverse: GCGCCGGAAAGCTGTAGAT
*GAPDH*	Forward: CTTTTGCGTCGCCAGCCGAG Reverse: GCCCAATACGACCAAATCCGTTGACT

## Data Availability

Not applicable.

## Sample Availability

Samples of the compounds are not available from the authors.

## References

[1] WHO Cardiovascular Diseases (CVDs). https://www.who.int/news-room/fact-sheets/detail/cardiovascular-diseases-.

[2] Flynn M.C., Pernes G., Lee M.K.S., Nagareddy P.R., Murphy A.J. (2019). Monocytes, macrophages, and metabolic disease in atherosclerosis. Front. Pharmacol..

[3] Ley K., Laudanna C., Cybulsky M.I., Nourshargh S. (2007). Getting to the site of inflammation: The leukocyte adhesion cascade updated. Nat. Rev. Immunol..

[4] Lasky L.A. (1995). Selectin-carbohydrate interactions and the initiation of the inflammatory response. Annu. Rev. Biochem..

[5] Wedepohl S., Dernedde J., Vahedi-Faridi A., Tauber R., Saenger W., Bulut H. (2017). Reducing Macro- and Microheterogeneity of N-Glycans Enables the Crystal Structure of the Lectin and EGF-Like Domains of Human L-Selectin To Be Solved at 1.9 Å Resolution. ChemBioChem.

[6] Adrielle Lima Vieira R., Nascimento de Freitas R., Volp A.C.P. (2014). Adhesion molecules and chemokines; relation to anthropometric, body composition, biochemical and dietary variables. Nutr. Hosp..

[7] Szabó-Fodor J., Bónai A., Bóta B., Szommerné Egyed L., Lakatos F., Pápai G., Zsolnai A., Glávits R., Horvatovich K., Kovács M. (2017). Physiological Effects of Whey- and Milk-Based Probiotic Yogurt in Rats. Polish J. Microbiol..

[8] Zhong Q., Wei B., Wang S., Ke S., Chen J., Zhang H., Wang H. (2019). The Antioxidant Activity of Polysaccharides Derived from Marine Organisms: An Overview. Mar. Drugs.

[9] Takahashi M., Takahashi K., Abe S., Yamada K., Suzuki M., Masahisa M., Endo M., Abe K., Inoue R., Hoshi H. (2020). Improvement of Psoriasis by Alteration of the Gut Environment by Oral Administration of Fucoidan from Cladosiphon Okamuranus. Mar. Drugs.

[10] Huang L., Shen M., Morris G.A., Xie J. (2019). Sulfated polysaccharides: Immunomodulation and signaling mechanisms. Trends Food Sci. Technol..

[11] Gacesa P. (1988). Alginates. Carbohydr. Polym..

[12] Bouissil S., El Alaoui-Talibi Z., Pierre G., Michaud P., El Modafar C., Delattre C. (2020). Use of Alginate Extracted from Moroccan Brown Algae to Stimulate Natural Defense in Date Palm Roots. Molecules.

[13] Zayed A., El-Aasr M., Ibrahim A.-R.S., Ulber R. (2020). Fucoidan Characterization: Determination of Purity and Physicochemical and Chemical Properties. Mar. Drugs.

[14] Li B., Lu F., Wei X., Zhao R. (2008). Fucoidan: Structure and bioactivity. Molecules.

[15] Liu J., Wu S.-Y., Chen L., Li Q.-J., Shen Y.-Z., Jin L., Zhang X., Chen P.-C., Wu M.-J., Choi J. (2020). Different extraction methods bring about distinct physicochemical properties and antioxidant activities of Sargassum fusiforme fucoidans. Int. J. Biol. Macromol..

[16] Chollet L., Saboural P., Chauvierre C., Villemin J.-N., Letourneur D., Chaubet F. (2016). Fucoidans in Nanomedicine. Mar. Drugs.

[17] Ahmad T., Eapen M.S., Ishaq M., Park A.Y., Karpiniec S.S., Stringer D.N., Sohal S.S., Fitton J.H., Guven N., Caruso V. (2021). Anti-Inflammatory Activity of Fucoidan Extracts In Vitro. Mar. Drugs.

[18] Lee H., Kim J.-S., Kim E. (2012). Fucoidan from Seaweed Fucus vesiculosus Inhibits Migration and Invasion of Human Lung Cancer Cell via PI3K-Akt-mTOR Pathways. PLoS ONE.

[19] Moumbock A.F.A., Li J., Mishra P., Gao M., Günther S. (2019). Current computational methods for predicting protein interactions of natural products. Comput. Struct. Biotechnol. J..

[20] Chen L.-M., Tseng H.-Y., Chen Y.-A., Tanzih A., Haq A., Hwang P.-A., Hsu H.-L. (2020). Oligo-Fucoidan Prevents M2 Macrophage Differentiation and HCT116 Tumor Progression. Cancers.

[21] Park J., Yeom M., Hahm D.H. (2016). Fucoidan improves serum lipid levels and atherosclerosis through hepatic SREBP-2-mediated regulation. J. Pharmacol. Sci..

[22] Sun J., Sun J., Song B., Zhang L., Shao Q., Liu Y., Yuan D., Zhang Y., Qu X. (2016). Fucoidan inhibits CCL22 production through NF-κB pathway in M2 macrophages: A potential therapeutic strategy for cancer. Sci. Rep..

[23] Simon S.I., Chambers J.D., Butcher E., Sklar L.A. (1992). Neutrophil aggregation is beta 2-integrin- and L-selectin-dependent in blood and isolated cells. J. Immunol..

[24] Bargatze R.F., Kurk S., Butcher E.C., Jutila M.A. (1994). Neutrophils roll on adherent neutrophils bound to cytokine-induced endothelial cells via L-selectin on the rolling cells. J. Exp. Med..

[25] Kansas G.S. (1996). Selectins and their ligands: Current concepts and controversies. Blood.

[26] Pouyani T., Seed B. (1995). PSGL-1 recognition of P-selectin is controlled by a tyrosine sulfation consensus at the PSGL-1 amino terminus. Cell.

[27] Sako D., Comess K.M., Barone K.M., Camphausen R.T., Cumming D.A., Shaw G.D. (1995). A sulfated peptide segment at the amino terminus of PSGL-1 is critical for P-selectin binding. Cell.

[28] Hidalgo A., Peired A.J., Wild M.K., Vestweber D., Frenette P.S. (2007). Complete Identification of E-Selectin Ligands on Neutrophils Reveals Distinct Functions of PSGL-1, ESL-1, and CD44. Immunity.

[29] Huma Z.E., Sanchez J., Lim H.D., Bridgford J.L., Huang C., Parker B.J., Pazhamalil J.G., Porebski B.T., Pfleger K.D.G., Lane J.R. (2017). Key determinants of selective binding and activation by the monocyte chemoattractant proteins at the chemokine receptor CCR2. Sci. Signal..

[30] Ritchie T.J., Macdonald S.J.F., Peace S., Pickett S.D., Luscombe C.N. (2013). Increasing small molecule drug developability in sub-optimal chemical space. Medchemcomm.

[31] Crijns H., Adyns L., Ganseman E., Cambier S., Vandekerckhove E., Pörtner N., Vanbrabant L., Struyf S., Gerlza T., Kungl A. (2021). Affinity and Specificity for Binding to Glycosaminoglycans Can Be Tuned by Adapting Peptide Length and Sequence. Int. J. Mol. Sci..

[32] Lagorce D., Douguet D., Miteva M.A., Villoutreix B.O. (2017). Computational analysis of calculated physicochemical and ADMET properties of protein-protein interaction inhibitors. Sci. Rep..

[33] Bernimoulin M.P., Zeng X.-L., Abbal C., Giraud S., Martinez M., Michielin O., Schapira M., Spertini O. (2003). Molecular Basis of Leukocyte Rolling on PSGL-1. J. Biol. Chem..

[34] Waldron T.T., Springer T.A. (2009). Transmission of allostery through the lectin domain in selectin-mediated cell adhesion. Proc. Natl. Acad. Sci. USA.

[35] Cumashi A., Ushakova N.A., Preobrazhenskaya M.E., D’Incecco A., Piccoli A., Totani L., Tinari N., Morozevich G.E., Berman A.E., Bilan M.I. (2007). A comparative study of the anti-inflammatory, anticoagulant, antiangiogenic, and antiadhesive activities of nine different fucoidans from brown seaweeds. Glycobiology.

[36] Thorlacius H., Vollmar B., Seyfert U.T., Vestweber D., Menger M.D. (2000). The polysaccharide fucoidan inhibits microvascular thrombus formation independently from P- and l-selectin function in vivo. Eur. J. Clin. Investig..

[37] Smith B.A.H., Bertozzi C.R. (2021). The clinical impact of glycobiology: Targeting selectins, Siglecs and mammalian glycans. Nat. Rev. Drug Discov..

[38] Jarnagin K., Grunberger D., Mulkins M., Wong B., Hemmerich S., Paavola C., Bloom A., Bhakta S., Diehl F., Freedman R. (1999). Identification of Surface Residues of the Monocyte Chemotactic Protein 1 That Affect Signaling through the Receptor CCR2. Biochemistry.

[39] Hemmerich S., Paavola C., Bloom A., Bhakta S., Freedman R., Grunberger D., Krstenansky J., Lee S., McCarley D., Mulkins M. (1999). Identification of Residues in the Monocyte Chemotactic Protein-1 That Contact the MCP-1 Receptor, CCR2. Biochemistry.

[40] Joshi N., Tripathi D.K., Nagar N., Poluri K.M. (2021). Hydroxyl Groups on Annular Ring-B Dictate the Affinities of Flavonol–CCL2 Chemokine Binding Interactions. ACS Omega.

[41] Yu X.-H., Zhang J., Zheng X.-L., Yang Y.-H., Tang C.-K. (2015). Interferon-γ in foam cell formation and progression of atherosclerosis. Clin. Chim. Acta.

[42] Lee J.-O., Bankston L.A., Robert C., Liddington M.A.A. (1995). Two conformations of the integrin A-domain (I-domain): A pathway for activation?. Structure.

[43] Shimaoka M., Xiao T., Liu J.-H., Yang Y., Dong Y., Jun C.-D., McCormack A., Zhang R., Joachimiak A., Takagi J. (2003). Structures of the alpha L I domain and its complex with ICAM-1 reveal a shape-shifting pathway for integrin regulation. Cell.

[44] Edwards C.P., Fisher K.L., Presta L.G., Bodary S.C. (1998). Mapping the Intercellular Adhesion Molecule-1 and -2 Binding Site on the Inserted Domain of Leukocyte Function-associated Antigen-1. J. Biol. Chem..

[45] Fisher K.L., Lu J., Riddle L., Kim K.J., Presta L.G., Bodary S.C. (1997). Identification of the binding site in intercellular adhesion molecule 1 for its receptor, leukocyte function-associated antigen 1. Mol. Biol. Cell.

[46] Rowe A., Berendt A.R., Marsh K., Newbold C.I. (1994). Plasmodium falciparum: A Family of Sulfated Glycoconjugates Disrupts Erythrocyte Rosettes. Exp. Parasitol..

[47] Skidmore M.A., Mustaffa K.M.F., Cooper L.C., Guimond S.E., Yates E.A., Craig A.G. (2017). A semi-synthetic glycosaminoglycan analogue inhibits and reverses Plasmodium falciparum cytoadherence. PLoS ONE.

[48] Szklarczyk D., Gable A.L., Nastou K.C., Lyon D., Kirsch R., Pyysalo S., Doncheva N.T., Legeay M., Fang T., Bork P. (2021). The STRING database in 2021: Customizable protein–protein networks, and functional characterization of user-uploaded gene/measurement sets. Nucleic Acids Res..

[49] Daina A., Michielin O., Zoete V. (2019). SwissTargetPrediction: Updated data and new features for efficient prediction of protein targets of small molecules. Nucleic Acids Res..

[50] Daina A., Michielin O., Zoete V. (2017). SwissADME: A free web tool to evaluate pharmacokinetics, drug-likeness and medicinal chemistry friendliness of small molecules. Sci. Rep..

[51] Bella J., Kolatkar P.R., Marlor C.W., Greve J.M., Rossmann M.G. (1998). The structure of the two amino-terminal domains of human ICAM-1 suggests how it functions as a rhinovirus receptor and as an LFA-1 integrin ligand. Proc. Natl. Acad. Sci. USA.

[52] Lubkowski J., Bujacz G., Boqué L., Peter J.D., Tracy M.H., Alexander W. (1997). The Structure of MC P-1 in Two Crystal Forms Provides a Rare Example of Variable Quaternary Interactions. Nat. Struct. Biol..

[53] Morris G.M., Huey R., Lindstrom W., Sanner M.F., Belew R.K., Goodsell D.S., Olson A.J. (2009). AutoDock4 and AutoDockTools4: Automated docking with selective receptor flexibility. J. Comput. Chem..

[54] Ramírez D., Caballero J. (2018). Is It Reliable to Take the Molecular Docking Top Scoring Position as the Best Solution without Considering Available Structural Data?. Molecules.

[55] Yurdakok Dikmen B., Alpay M., Kismali G., Filazi A., Kuzukiran O., Sireli U.T. (2015). In Vitro Effects of Phthalate Mixtures on Colorectal Adenocarcinoma Cell Lines. J. Environ. Pathol. Toxicol. Oncol..

[56] Moss J.W.E., Davies T.S., Garaiova I., Plummer S.F., Michael D.R., Ramji D.P. (2016). A Unique Combination of Nutritionally Active Ingredients Can Prevent Several Key Processes Associated with Atherosclerosis In Vitro. PLoS ONE.

